# Thinking on the purposes, roles and activities of networks for research on maternal and perinatal health: opinions of coordinators and members

**DOI:** 10.61622/rbgo/2024rbgo75

**Published:** 2024-09-06

**Authors:** Vilma Zotareli, Silvana Bento, Renato Souza, José Guilherme Cecatti

**Affiliations:** 1 Department of Obstetrics and Gynecology Universidade Estadual de Campinas Campinas SP Brazil Department of Obstetrics and Gynecology, Universidade Estadual de Campinas, Campinas, SP, Brazil.; 2 Center for Research in Reproductive Health Campinas SP Brazil Center for Research in Reproductive Health, Campinas, SP, Brazil.

**Keywords:** Networking, Goals, Leadership, Motivation, Interview, Perinatal care, Reproductive health

## Abstract

**Objective:**

To identify the opinion of coordinators and members about the essential characteristics and to understand the research networks characteristics, to facilitate their implementation, sustainability and effectiveness so it can be replicated in low and middle-income countries.

**Methods:**

A qualitative study using a semi-structured interview technique was conducted. We selected potential members, managers and participants of networks from publications identified in PubMed. After checking the FIGO congress program, we identified authors who were assigned as speakers at the event. An invitation was sent and interviews were scheduled.

**Results:**

In total, eleven interviews were performed. Coordinators and members of networks have the same goal when they decide to participate in a network. In general, they cited that these individuals had to be committed, responsible and enthusiastic people. The network should be composed also of postgraduate students. A network should allow multi-leadership, co-responsibility, autonomy and empowerment of its members. Effective communication was mentioned as an important pillar for network maintenance. Another motivation is being an author or coauthor in publications. One way to maintain a network running is social or governmental commitment, after resources expire, studies continue.

**Conclusion:**

Networks are different due to the social context where they are inserted, however, some characteristics are common to all of them, such as having engaged leaders. For an effective and sustainable network, commitment and motivation in a leader and members are more in need than financial resources. Ideally, to ensure the operation of the network, the institution where the leader is linked should support this network.

## Introduction

High-quality research that produces impactful scientific knowledge involves creating reasonable and objective research questions, developing appropriate study designs and using robust and reliable data. The collaboration between researchers, institutions and countries has facilitated this process, promoting discussion on priority agenda, fund raising, knowledge exchange and sustainability in the research area. The phenomenon of research collaboration can be considered a network, depending on some characteristics of collaboration. However, the establishment of essential network characteristics and fundamental properties for its success remains uncertain.^1-3^ A better understanding of these characteristics may facilitate research network implementation, especially in low and middle-income countries, where a fragmented scientific community is common.^4^

Network could be defined as “a group of people who exchange information and contacts for professional or social purposes” with an open structure that is able to expand and communicate, in a dynamic and open way, susceptible to change without threatening its equilibrium.^5^Network comprises a set of autonomous members uniting ideas around shared values and interests within and across many areas of knowledge.^6,7^ Networking collaboration is advantageous to improve science. Continuous and organized collaboration facilitates the achievement of more challenging goals, saving funds and time when compared to individual attempts.^2^Impacting and translational scientific knowledge, when new science is translated to innovative health care policies, and human resource training are other benefits associated with the establishment of research networks.^1-4^

Researchers join around a networking group to produce robust and standardized studies, improving data quality aimed at acquiring translational cutting-edge knowledge. The participation of researchers and study subjects from common and different settings is of great interest to tackle relevant health problems, maximizing reproducibility, external validation, in addition to promoting capacity building of human and equipment resources and promoting an exchange of skills between professionals from different institutions.^1,8-12^The mentioned benefits also increase the chances that networks may raise funds for research projects and promote practices, attitudes and techniques of professionals benefitting patients and, more importantly, impacting society.^1,3,13,14^Network collaboration, especially in priority areas such as maternal health, is especially important. It can integrate small research groups from low- and middle-income countries with high-income countries and produce evidence for improvement in global health and health equity, as well as economic development.^4,14,15^

The World Health Organization considers maternal health a priority in research and public health care. An improvement in women’s health may have a positive impact on her family, community and health care system and result in better health for the next generations. Since the 90’s, a decrease in the maternal mortality ratio has been observed in many low, middle- and high-income countries and is the result of simple evidence-based solutions and inexpensive interventions.^16,17^Unexpansive interventions may not hold the same interest and attention of the industry and private health services. Despite being a priority, maternal health research lacks investments and funds when compared to other male and female health areas. Although effective interventions are available, progress may have been limited in this area.^16,17^The WHO, however, has worked to reduce maternal mortality by increasing research, evidence and technical support to technicians and physicians. In addition, it has set a global standard index and stimulates research networks in the area.^16^

Some networks have been established in a search for evidence based-solutions and strategies aligned with priority agendas, similar to that established by the WHO. For example, the Global Obstetrics Network (GoNet) states that its “*purpose is to foster communication between groups to improve ongoing and future trials. This will open new avenues for cooperation in the design and conduct of large international trials, in seeking funding, and in highlighting evidence. It is expected that this will lead to better studies, a more efficient use of resources and minimize duplication*”.^18^Difficulties reported by the group include sharing information about all projects conducted by all members and proper communication with all the different centers, which could be a limitation if not all researchers have a good command of English. The International Network of Obstetric Survey Systems (INOSS), a multi-country network for the study of uncommon and severe complications of pregnancy and childbirth, emphasizes the benefits of networking, enabling standardized data collection on rare conditions with very low rates in pregnancy.^19^

A collaborative initiative can emerge through different manners: researchers, funders, stakeholders, policy-makers, governments. GONet and INOSS for instance emerged from researchers.^18,19^On the other hand, the Adolescent Medicine Trials Network for HIV/AIDS, an independent and collaborative research initiative that explores promising behavioral, microbicidal, prophylactic, therapeutic, and vaccine modalities in HIV-infected and at-risk youths emerged from the National Institutes of Health (NIH) of the USA Government. This initiative aims to stimulate the engagement of junior investigators with fresh perspectives and innovative ideas, promoting progress in this specific field.^20^

Networks have different characteristics of conception, purpose, organization, objectives and formalities. However, it remains unclear whether there are core essential characteristics that are crucial for the sustainability and success of a network. The lack of understanding of whether or not heterogeneity occurs when characterizing research networks, makes it more difficult to identify relevant factors for the establishment of networks and characterization of “success” factors. Therefore, we aimed to characterize core essential characteristics and understand research networks to facilitate its implementation, sustainability and effectiveness, in order to replicate the network model, especially in low- and middle-income countries.

## Methods

A qualitative study was conducted using a semi-structured interview technique during the International Federation of Obstetrics and Gynecology – FIGO World Congress of Gynecology and Obstetrics held in Rio de Janeiro, Brazil, in October 2018.

**Figure 1:**
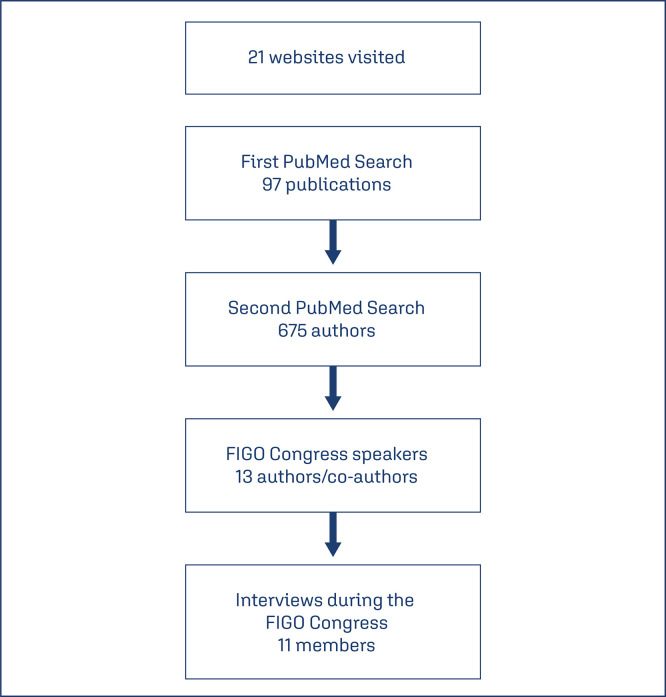
Members identification and interviews

In order to identify the members, first of all, a search in PubMed was done using the terms “Reproductive Health”, “Network” and “Consortium” to retrieve information on potential networks in this field. Throughout this search, five networks were identified. Later, a new search was carried out in PubMed to identify members of these networks who were authors or co-authors of the articles related to the networks´ studies. In this process, 675 authors were identified in 97 articles. For the names identified, we looked for the FIGO Congress scientific program if there were some names on the program. Thirteen authors were assigned as speakers at the event. They were invited and 12 accepted to participate in this study. One did not answer the invitation. Among 12 authors who accepted to participate, one did not travel to Brazil to attend the scientific event due to personal problems. So, 11 interviews were carried out with this convenience sample, including 6 coordinators and 5 members of networks (Figure 1).

We sent an invitation letter explaining the research and invited them to participate in it three months prior to the event. If they agreed to participate in the study, an interview during the event was scheduled according to the convenience of the participant. The interviews were conducted individually by two investigators in a private room. The interviews were done in English, Spanish or Portuguese. All interviews were recorded. On average, it took around 23 minutes to complete each interview, with a minimum of 19 minutes and a maximum of 59 minutes. The guide for the interviews was divided into 1) Process to establish the Networking group, including motivation, actions, initial difficulties accepting the process of members, and refusal; 2) Number of members, characteristics of the members, motivation to join and stay in the group; 3) Personal evaluation of the group, publications, results, facilities, difficulties in maintaining its operation; 4) Aspects which could be improved in the current networking group such as dissemination of results, communication with members, empowerment of leaders; 5) Characteristics which could be considered important to keep the group working such as horizontality, multi-leadership, co-responsibility, autonomy and empowerment of the members, free entrance and exit. To carry out the semi-structured interviews a specific guide was prepared and pre-tested with four professionals who have the same characteristics as the members of this study.^21^ For data analysis, the Patton guidelines were followed.^22^First, the transcriptions were read, and units of meaning were marked in the speech of members, according to study objectives. Six members live/work in countries which are classified by the Human Development Index.^23^between 0.7 and 0.8, considered a high index. Categories for analysis were created from the units of meaning. These categories were composed of codes applied to portions of the text and later similar passages were grouped together by category in all interviews. Afterwards, a content analysis of each group of texts was performed, based on the proposed categories of analysis and in the study objectives. In this article, we present an analysis of the following categories: a) composition of networks; b) performance of networks; c) motivation to participate in the networks, d) difficulties in network implementation; and e) challenges of network sustainability (Chart 1).

**Chart 1:** Categories and subcategories studied

	Categories	Subcategories
a	Composition of networking	characteristics of coordinators characteristics of members characteristics of networking
b	Performance of networking	freedom responsibility rules permanent professionals
c	Motivation to participate in the networking	believe in the proposal of networking improve professional qualifications publications networking presential meetings new research projects written rules communication
d	Difficulties in networking implementation	participating centers research database financial resources limited budget payment for members
e	Challenges of networking sustainability	keeping young researchers financial resources continuity of network

The Institutional Review Board of the University of Campinas approved the study protocol (No. 2.825.315/2018) (*Certificado de Apresentação de Apreciação Ética*: 84398018.4.0000.5404). Each participant signed an informed consent form before starting the interview.

## Results

Among the 11 people interviewed, 6 were coordinators and 5 members of networks. All of them belong to 8 different Maternal and Perinatal Health Networks, located in different countries (Table 1). Regarding the characteristics of the participants, the majority were physicians, most of them coordinators of a network and also members from other ones. Thus, three members have been working in the network for 11-15 years, two for 16-20 years and six for more than twenty years. Among the coordinators, five of them have been working as coordinators for more than 10 years. Three from very high classification (>0.8) and two from middle/low countries (<0.7) (Table 1). In the presentation of the results, the coordinators of networks were identified as “CO” plus a number; the members were identified as “ME” followed by a number.

**Table 1:** Characteristics of members in the study (n = 11)

Characteristics	n
Gender	
Male	7
Female	4
Role in network	
Coordinator	6
Member	5
Profession	
Physician	10
Other	1
Time working in the networking	
01 - 15 years	3
16 - 20 years	2
< 20 years	6
Country where live according to HDI	
Very high > 0.8	3
High 0.7 - 0.8	6
Middle/low < 0.7	2
Type of networking	
National	4
International	7

*All the coordinators acting also as members of other networking

### Composition of networkings

#### Characteristics of the coordinators

The network coordinator should be a prominent health professional who contributes to “scientific production” in a relevant manner. The coordinator should also be able to obtain funding for research development and establishing links with an institution that has a scientific community reputed for its excellence may also influence the work of the members. This person should be motivated and committed, as well as a skilled communicator. The other skills are to be a good listener, be argumentative, and speak English. These personal characteristics of the coordinator inspire the respect of the members, which is necessary since the coordinator plays the role of network “aggregator.”

*The first is the personal aspect and the respect that people have for the coordinator, an extremely important person for scientific production at a national level. […] the respect for the institution and the coordinator is an aggregating factor. (ME 01)*

#### Characteristics of the members

Network members must be motivated and enthusiastic individuals. They should aspire to learn with the study and wish to publish articles. Members should have the political support to make the study viable in the institution where they are linked and overcome obstacles. They must bear in mind that “the group prevails and not the individual” and believe in the topic of study. A coordinator reported that a researcher should be a “serving leader” because “he is there to serve the group, and not there to be promoted... the higher good is the network, the project”. It should be clear that what the member is doing in the research is important, that only he can execute the task, and that only this research group can accomplish this. A network should also be composed of postgraduate students, interested in improving their studies and pursuing an academic career. Senior researchers should be included as well.

*The leader who serves the network achieves greater prominence due to network survival than for personal recognition [...]. (CO 03)*

#### Characteristics of networking

A network should be composed of more developed and less developed institutions and members with varying experience in the development of research projects. There should be student members and some senior members. There was a consensus among members that this merger provides greater knowledge to all involved and a more significant accomplishment of network tasks. There is a permanent professional group it is important to improve the networking performance. Once, those professionals work together in different projects they become experts doing it.

*Have a number of junior (PhD Students) and senior person to give them advice. If you are forming a network, from every university you must have maybe one or two seniors, but three or four juniors [...]. (CO 05)*

#### Performance of networkings

Both categories of members consider that networks should have multileadership, co-responsibility, autonomy and empowerment. These characteristics have been shown to be interwoven in routine network practice.

#### Freedom

It was considered that all network members should have the freedom to voice their opinions, propose new projects in the network and also make some decisions about ongoing studies along with the group. Furthermore, network coordination “must permit local initiatives to promote the project. The autonomy of its members is thus stimulated.

#### Responsibility

The responsibility for the studies developed in the network should be of all the researchers who compose the network. Co-responsibility was considered a pillar of the network by the members because each one feels responsible for what he does and therefore is more dedicated to network tasks. Nevertheless, there should be only one leader, who is the coordinator. Empowerment of members should be encouraged so that they can perform the work, not because the coordinator requested, but because they consider it necessary for the network as a whole. Network tasks should be highlighted and not individual work performed by its members. In this scenario, they believe that full horizontality in the network cannot exist, because in practice a leadership “that stands out a little” is necessary.

*If the people do not play their parts (in the project, in the network), it does not happen. (CO 04)*

#### Rules

Rules should be transparent for the consolidation of the network. Some coordinators reported that in their network there were written rules on management of the database of research studies and also on the authorship of scientific articles. Others stated that there was no need for written rules because there is already a variety of bureaucracy to follow in research.

*Transparency is important, there can be no close-knit groups, and no favoritism towards friends, it has to be something really transparent based on rules. – transparency in communication so everyone knows that they received the same treatment. (CO 03)*

#### Permanent professionals

Another necessary aspect of ideal network operation was to have permanent professionals working in administrative support. This support included a manager and administrative staff available. Having a permanent staff was considered a facilitator for work performance. Permanent workers in a network acquire experience in various research studies and work is improved.

## Motivation to participate in the networkings

### Believe in the proposal of the networks

Coordinators and members of a network have the same goals when they decide to participate in a network, which are: investigate parameters that seek to reduce maternal mortality, acquire knowledge of new research topics, believe in the theme proposed for the research study; public policy actions; interact with researchers from other institutions. Creation of an implementation agenda of public policy actions to present the results found in the studies and discussed with the health authorities to make them available in the health care area. This all seems to strengthen researchers individually.

### Improve professional qualifications

Participation in a research network can bring benefits to both the researcher and the institution where he is linked. For researchers it was considered a promoter of academic and professional development. In the academic setting, the network promotes and stimulates its members to exchange ideas with participating centres. It also encourages the members to qualify and create bonds with reputed international institutions and improve their knowledge by interacting with younger members, such as postgraduate students and senior professionals. In the professional setting, participation in a network may boost a professional academic career since many network members after contact with research and working in a university setting enhance their knowledge and increase their scientific publications. On the other hand, the participating centre has more prepared professionals who may use the data collected in research studies developed through the network for their own studies and also for student guidance. The importance of an exchange program of professionals between institutions to strengthen the postgraduate course was mentioned by the members.

*The fact that we are related to the network and can go on an exchange program strengthens our postgraduate course. (ME 03)*

Furthermore, qualified people are influenced to write research projects and generate new publications.

### Publications

Being a co-author in publications motivates members to wish to be part or continue working in a network, especially young researchers.

*Publication is a very important ingredient, because the people want to advance in the career. And the only way to advance in university is to publish. (CO 05)*

### Networking meetings

Face-to-face meetings are necessary to improve communication and integration between network members. Meetings with network members should be held periodically. There was no consensus as to periodicity, but ideally, an initial meeting and a final meeting concerning developing studies should take place.

*Important if it can raise the money to do it at least once a year [ ...]. It is good to do it face to face. (CO 05)*

An obstacle to these meetings is the lack of financial resources. Someone suggested that meetings could take place during scientific events. The reason is that may network members participate in these events using personal resources or are funded by the institution where they are linked. Another alternative mentioned was holding online meetings and social media networks.

*We made massive online meetings, like in a video conference online where everyone was able to connect and we revised the protocol with everybody [...]. (CO 03)*

### Written rules

There was a lack of consensus among coordinators concerning written rules. Networks that had this document reported that these rules had been elaborated to *reduce tension between people, and relax since they knew that they actually had an agreement on this* (CO 03).

Network coordinators who had no written norms considered it unnecessary to have this document because research already has a bureaucracy to follow.

### Communication

Agile and effective communication should be maintained between network members. Close ties between researchers may be established, achieving problem resolution, and causing a positive impact on research development. Communication can be maintained by electronic address (e-mail) messages, telephone, WhatsApp and social media networks. Furthermore, an informative newsletter may also be produced. Members also agreed on this topic. Great allies in this process are the social media networks that enable rapid and effective communication. Nevertheless, routine network practice is faced with an obstacle to maintaining efficient, transparent and continuous communication, due to the lack of professionals for this activity. In practice, this activity is delegated to a researcher, for example, a physician who has many other activities to perform. Due to the demands in his work schedule, he may be unable to maintain rapid and efficient communication, hindering the motivation of the members and consequently research development. Ideally, a specific professional should perform this task.

*making phone calls, showing that the group is important. So, if you do not have a good manager the network is difficult to maintain. (CO 06)*

### Difficulties in networking implementation

Both members and coordinators mentioned some topics identified as difficulties in network implementation. Coordinators reported some problems that occurred during the development of some studies, as well as the way these difficulties were resolved.

### Participating centers

It was mentioned that the work rate of the participating centres was not uniform concerning data collection. Several factors are involved to perform this task, such as ethical approval, effort invested by the researchers, etc. A strategy used by coordinators was to grade participating centres for their performance in data collection. Grading was suggested because there was a very high inequality among centres. Some had begun data collection, while others had not even started, despite a lack of an apparent reason. Centres that had not collected data were given a grade of zero. This made researchers angry, although, after a few months, all centres had recruited members. Another reported that when a centre is unable to collect data even after receiving a visit from the coordinator, researchers are dismissed from the study. There is a time frame for data collection and the task involves expenses using financial resources. Members commented on the difficulties in maintaining a greater integration between the coordinating centre and participating centres.

### Research database

In general, data collected in research are stored in one site under the responsibility of the institution that coordinates the network. Some coordinators reported that after research ends and articles have been written, data on participating centres are sent to local researchers. Therefore, in practice, these researchers do not have these data in hand. Some researchers ask the network coordinating centre for help in writing articles.

### Financial resources

To maintain a structured network there must be funding for research projects. The budget for projects includes resources for payment of professionals working in administrative, statistical, construction support and site maintenance of the network. According to the majority of study members, in practice, researchers linked to network coordination exert these functions and consequently neglect some due to a lack of time. With resources to pay for/hire a professional, tasks would be easier to execute.

*We have administrative support, You need to have it. We need a manager, administrative personnel. (CO 06)*

### Limited budget

Another difficulty mentioned is that there is a limited budget for research. Participating centers receive modest sums that are predicted within budget. Should any problem occur that prolongs completion of the study, no money will be left to pay for the research study. There is discrimination among international assistance agencies against researchers from developing countries. These researchers need partners in more developed countries to obtain funding for large projects.

### Payment for members

Coordinators did not reach a consensus over payment of network members for their work. Some defended monetary compensation, albeit symbolic, while others thought that the motivation to participate in the network cannot be financial. There is difficulty in funding the many centres.

*It would be unfair to invite people from a low-income country to work with you in projects, asking them to invest time, have duties with no monetary compensation [...] somebody has to pay for that. (CO 06)*

On the other hand, interviewees who had considered paying researchers unnecessary, said that the person did not have to receive a salary, but a symbolic form of payment. Other incentives would be to offer these researchers co-authorship in articles and the network could fund their participation in scientific events, for example:

*In the grant you have to put others [...]. Obviously, people do more than what the grant says [...] they ask me more than salary sometimes. (CO 05)*

## Challenges of networkings sustainability

### Keeping young researchers

To bring a young professional to work with you and the remaining group is a remarkable, successful feat of the network that will ensure its continuity.

*if you are able to bring a junior, who would work with you and then also sit in the group as a full member, I think that is a big achievement, and warranty the continuous network activities. (CO 05)*

*It started with residence training and after that, I was part of research groups, for twenty years. I think, it is the people who are involved, committed to doing something good. (CO 04)*

### New research projects

New research projects with necessary funding are required to maintain a network running.

*Keep them motivated, you do not let the group die. (CO 06)*

### Financial resources

It was reported that every project needs financial support for study development. To maintain a network active, one solution was to have active micro-networks working on projects with a low budget until an excellent opportunity arises. Another important aspect is to look for information on foreign policies to see where funding is allocated. The researcher can seek centres and researchers of prioritized countries and ask to be part of the network.

*[...] that´s a more political thing [...] you really have to think about who am I choosing for getting money [...]. (CO 05)*

### Continuity of the network

Maintaining a network alive depends on the support of teaching institutions and also governmental organs. A way to maintain a network in operation is social or governmental commitment, so that after resources expire, research studies continue. Governmental authorities need to understand the importance of intervention/research for women’s health and continue these activities. Another point was that when a project ends, the next project should already be proposed. Network coordination should stimulate this action.

## Discussion

We were able to observe that characterizing a research network is a complex process. However, diverse factors related to its conception, sustainability and success were shown to be homogeneous according to the professionals participating in this study. Thus, we were able to understand that despite different networks with different characteristics, simultaneously they also have characteristics in common that may be considered essential, including that all those involved should have a common purpose, a prominent leader, objective and effective communication and access to funding.

Moreover, this study does have some limitations. Potential members were selected by review of an international congress program. Therefore, only known network coordinators and members attending the congress were invited to participate in the study. In addition, the invitation was sent only to potential network members taking part in the congress agenda. Speakers at scientific events are usually more experienced researchers and likely to be network coordinators. Most of the members of a few networks, which is also a limitation. Despite these limitations, this qualitative study allows us to discuss some relevant aspects for the good functioning and sustainability of research networks, according to the opinion of professionals who participate in them.

Professionals are linked to a research network because they can relate to their topic of interest. In this study, the topic was reproductive and perinatal health.^5^Various networks were created with distinct goals, such as the study of rare diseases in pregnancy, and the publication of outcomes of large international clinical trials, among others. Some appeared due to the demand of researchers and others from the need identified by governmental public organs.^18-20^Irrespective of this, however, the main purpose of a network is to make new discoveries and positively impact the health of the population, most specifically in women’s management and health care.^24^Networks are formed by individuals that are agents of an action, i.e., people act for a reason, according to their interests and experiences. In analogy, collaborators met to exert in a useful and efficient manner research in their field of knowledge, to produce and disseminate new knowledge for the area. Still, according to human relations theory by Mayo,^25^researchers who identify study aims in the networks that may strengthen their individual interests and satisfaction, become more cooperative and contribute more to the purposes and proposals of the network in a collective manner.

The leader appears to have a major role in this working gear and was a convergence point among coordinators and network members. The main role of the leader appears to be to stimulate and organize members to continue to be motivated and active in concluding their aims. For this to happen, however, the coordinator must use some strategies, such as objective and effective communication, so that everyone can understand their roles in the network and feel validated and motivated. Therefore, we observed that the leader coordinated network members based primarily on common goals. This leadership emerges from the recognition and legitimacy of the coordinator by the members. Our results either converge with or diverge from Weber’s theory.^26^There is convergence concerning the definition of power and authority. According to this sociologist, power is the capacity to influence the behavior of another person and authority is the acquired right to exert this influence within a group. Nevertheless, divergence occurs in settings defined by Weber, who states that authority is exerted by coercion, manipulation, or established norms.^26^In this manner, the theory of Mayo emphasizes that the individuals identify themselves with the network objectives and also get satisfaction when reach the proposed objectives; the need for reciprocal communication between leader and members and the development of a leader who communicates the aims to ensure effectiveness are essential elements.^25^In the case of study networks on reproductive and perinatal health, communication seems to have a fundamental role and is a common feature for all, not only the network coordinator but its members as well. When everyone has a clear understanding of the aim and where they want to go, actions become easier. This clarity is one of the fruits of good communication.^27^

A factor related to network sustainability was access to funding. Alternatives created by the network, mainly by the coordinators, seem to make the maintenance and coordination of a network more viable. Partnership with institutions and universities where members are linked is an alternative. The institution, acknowledging the importance of the work performed in the network and the repercussions on the health improvement of the population, provides human and financial resources, along with other types of support for network operation. Another alternative is to use low-cost tools such as the media and social media networks for communication and publicity, in addition to network meetings at national and international scientific events. Seeking alternatives to funding and other resources, therefore, was fundamental for network maintenance.

One challenge of network sustainability lies in achieving the permanence of younger researchers in the network, approval of funding and support of teaching and governmental institutions.

## Conclusion

According to coordinators and members of research networks, the researchers looking to participate in network research groups according to the objective of the research which they are interested in. Networks are different due to the social context where they are inserted, however, some characteristics are common to all of them, such as a leader and members that are committed and motivated, more than financial resources. Institutional support is fundamental to ensure these characteristics.
